# Proof of concept of a sexual health outreach program led by community health workers in homeless hostels in the greater Paris region

**DOI:** 10.3389/fpubh.2023.1305874

**Published:** 2024-01-12

**Authors:** Emma Vaugoyeau, Lison Rambliere, Manon David, Hanaa Lemguarni, Sylvie Le Gac, Armelle Pasquet-Cadre, Samy Rasli, Jade Ghosn, Willy Rozenbaum, Elisabeth Bouvet, Maëlle Prioux

**Affiliations:** ^1^Pôle DELTA, Samusocial de Paris, Paris, France; ^2^Observatoire du Samusocial de Paris, Samusocial de Paris, Paris, France; ^3^Pôle médical et soins, Samusocial de Paris, Paris, France; ^4^COREVIH Île-de-France Nord, Paris, France; ^5^COREVIH Île-de-France Est, Paris, France

**Keywords:** community health workers (CHW), precarity, outreach, sexual health, screening, homeless

## Abstract

**Context:**

Homeless individuals face exacerbated risks of infectious diseases, including sexually transmitted infections (STIs). Programs led by Community Health Workers (CHWs) have demonstrated potential to enhance healthcare access for marginalized groups such as homeless families. This study aims to evaluate the feasibility and effectiveness of a novel CHW-based outreach program addressing sexual health issues among individuals residing in homeless hostels.

**Methods:**

Twelve social homeless hostels in the greater Paris region were selected as program implementation sites. An outreach program was developed consisting of two interventions: sexual health workshops and STI screening sessions (HIV and hepatitis B and C) accompanied by individual interviews, both conducted by CHWs within each hostel over an 8-week period and scheduled weekly. Feasibility, participation and engagement were evaluated using complementary methods including qualitative field observations, semi-structured interviews and focus groups with CHWs, satisfaction questionnaires for participants, and quantitative outcome data collection of each intervention.

**Results:**

A total of 80 program activities (workshops and screening sessions) were conducted. Among the participants, 542 women and 30 men engaged in workshops. During the 30 Rapid Diagnostic Testing sessions, 150 individuals underwent testing for HIV, hepatitis B, and/or hepatitis C. Positivity rates were 6.7% for hepatitis B and 0.9% for hepatitis C. No HIV infections were detected. Participant satisfaction rates were consistently high (>76%) across workshops. Qualitative analysis unveiled two critical axes influencing program feasibility and effectiveness: program organization and CHW involvement.

**Discussion:**

This assessment of the program highlights its feasibility among a population that is difficult to reach through conventional healthcare efforts. The intervention’s potential effectiveness is suggested by self- and CHW-reported improvements in sexual health literacy and high rates of referral to the healthcare system, as well as holistic well-being considerations. CHW involvement is a vital determinant of program success, as are robust coordination among stakeholders, deep understanding of the target population, and strong partner engagement.

**Conclusion:**

This outreach program amplifies the voices of often-overlooked populations while empowering them to navigate health and social challenges. Although these workshops serve as lifelines for those frequently excluded from mainstream services, long-term improvements to the health and wellbeing of homeless populations will necessitate systemic governmental intervention.

## 1 Introduction

Homeless people experience poor living conditions, economic deprivation and sociocultural challenges that combine to have negative impacts on their physical, mental and sexual health (1). Women experiencing homelessness in France are predominantly foreign-born migrants, are at particularly high risk for sexually transmitted infections (STIs) such as HIV (2) and face risk factors such as sexual violence (3) and difficulties negotiating safer sex practices (4). Although homeless women are specifically targeted for STI screening, 31% report never having received an HIV test, with poorer access to screening among women with lower levels of education and French proficiency, as well as women in relationships (5). About a quarter of homeless women report unmet healthcare needs, but an even larger proportion report no healthcare needs whatsoever, even if their current health status is poor or they have specific risk factors (6, 7). Sexual health awareness actions therefore seem essential among this particularly vulnerable population.

Programs led by community healthcare workers (CHWs) have two common principles of intervention: “going toward” and “working with” people to strengthen their ability to make decisions favorable to their own health (8). Such outreach programs aim to empower some of society’s most isolated, precarious and/or vulnerable populations and are implemented outside clinical spaces, ideally where targeted populations are most likely to live or frequent (9). CHW programs aim to help individuals express their needs and expectations without discrimination or prejudice, while overcoming difficulties such as insecurity, language barriers, competitiveness with basic needs (i.e., food insecurity, housing instability, health), geographical and cultural distance from institutions, and poor understanding of the healthcare system (10–13). Ultimately, these programs are designed to help refer individuals to the healthcare system and to ensure their access to essential services such as health insurance and social assistance (11, 14, 15). In this way, CHW programs help to ensure respect for the human right to health, defined as the enjoyment of the highest attainable standard of health, to vulnerable population (16).

CHWs are defined by the World Health Organization (WHO) as health care providers that come from the community (14). They include a diversity of workers with heterogeneous profiles and training who can be name by other term according to the countries and populations (17). Their assignments, tasks, working environment, and remuneration are also highly dependent on the context of intervention (12). In France, CHWs are described as professional health workers or unpaid volunteers who commonly work in public health institutions, NGOs and social centers whether they come from the community or not (11, 14). CHWs are mainly recruited for their excellent knowledge of their territory, populations and local actors, their soft skills and sometimes for their similar experience of life or illness which they share with the community members (12).

The development of CHW programs in France is closely tied to the history of the HIV/AIDS crisis (18). Starting in the late 1980s, the challenges posed by the epidemic highlighted not just the medical and political system’s lack of control over the crisis, but also the influence of social inequalities on the spread and impact of HIV (10). CHW programs have been increasingly used by institutions and non-governmental organizations (NGOs) in recent years, including in Paris (19). In the greater Paris region, the most densely populated region in France with over 12 million inhabitants, around 1.8 million people live below the poverty line (828 euros/month). At the end of the 1990s, there was a sharp increase in the number of homeless women and families and isolated women in this region (20). Due to a shortage of available beds in shelters and social housing, many empty hostel rooms were repurposed as *homeless hostels* to provide rapid accommodation for this particularly vulnerable population (21). In 2021, over 850 homeless hostels housed more than 58,200 people every day in greater Paris (22). These people, mostly families, are housed for an average of two and a half years (10). Half are single mothers and the overwhelming majority were born outside France and live below the poverty line (23). They encounter many difficulties related to healthcare access, as a result of switching between different homeless hostels (24), with households moving from one hostel to another an average of five times during their stay (25).

Globally, evidence suggests that outreach programs involving CHWs can be effective for improving healthcare access in underserved or excluded groups with high non-utilization rates (15, 26, 27). In particular, by reducing barriers to accessing healthcare and addressing specific health needs in a culturally sensitive manner, these programs can help bridge the health equity gap (11–13, 15, 27–32). While the scientific literature on CHW programs in Low or Middle-Income Country (LMICs) is fairly dense, systematic reviews have highlighted important gaps in the literature in high-income countries, where the feasibility, efficacy and reproducibility of CHWs programs are rarely studied and/or published in peer-reviewed scientific journals (33, 34). The objectives of this study were to assess the feasibility and effectiveness of a novel CHW-led sexual health outreach program targeting individuals living in homeless hostels in the greater Paris region.

## 2 Program description

### 2.1 Program development

Prior to the development of our sexual health outreach program, a preliminary cross-sectional study was conducted by CHWs by discussing with individuals living in homeless hostels to identify their sexual health needs. This diagnostic phase took place in 2020 in three homeless hostels, with 6 CHWs from partner organizations working in the HIV sector, and aimed to identify the primary sexual health issues faced by the target population (predominantly women). These included a lack of knowledge about sexual health risks, challenges in accessing healthcare services, and a preference for non-mixed workshops for open discussions. Subsequently, the findings were used to design and implement the program.

All the 17 CHWs involved (referred to Box 1 for CHWs description) were invited to participate in working groups to co-develop the program and to define the program’s themes, tools to be used, organizational aspects such as scheduling, intervention days, and communication methods. To ensure consistency in the CHWs’ discourse and practices, training sessions were provided. These sessions focused on support to migrant individuals who have experienced violence, as well as the use of programs tools. During the study period, 12 homeless hostels were selected as implementation sites for the program. The selection process was based on several inclusion criteria, including being in the greater Paris region, having an accommodation capacity of over 100 individuals, and having a dedicated space available for program activities. In addition, priority was given to homeless hostels that rarely benefit from similar initiatives.

### 2.2 Intervention descriptions

The final outreach program consisted of engagement efforts to enroll participants and two distinct interventions: sexual health workshops and STI screening sessions.

To engage participants, 1 or 2 weeks prior to the launch of the outreach program, hostel personnel were asked to display posters in high-traffic areas (e.g., elevators, kitchens, and common rooms) to raise awareness among residents. One week prior to the first workshop, CHWs conducted door-to-door visits to meet each resident in their respective rooms, provide them with program-related information and distribute flyers. Contact details of interested residents were collected to send reminders via a dedicated WhatsApp group or text message.

Workshops and STI screening sessions were both conducted in all 12 homeless hostels over an 8-week period. They took place once a week for 3 h on a fixed day determined by the teams to allow participants to plan their attendance in advance and encourage consistent engagement.

Around six STI screening session workshops were held in the communal area of the hostel. In pairs, CHWs fostered a trusting atmosphere and introduced relevant topics related to sexual health. Four standardized tools were used: male and female anatomical charts with accompanying fill-in-the-blank captions for completion, a health promotion visual tool (35), a game (36) and an informational booklet containing a map with a comprehensive list of nearby healthcare services for potential referrals. Hygiene kits were also given to each participant with various products such as shampoo, shower gel, toothpaste, toothbrush, sanitary protection, and external condoms. Additionally, flyers were made available in multiple languages (French, English, Arabic, Pashto/Dari) that summarized the main sexual health subjects discussed and provided contact information for local sexual health organizations (37).

Following six workshop sessions, participants were given the opportunity to undergo two STI screening sessions. Voluntary screening for HIV and hepatitis B and C through Rapid Diagnostic Testing (RDT) were proposed. These screening sessions were led by the same CHWs who conducted the workshops. This screening process involved individual interviews conducted to gather socio-demographic information, identify risk-taking behaviors, and assess participant knowledge about STIs. Following the screening, participants were provided with an information sheet detailing their test results. If a participant was tested positive for at least one infection, they were referred to a hospital referral center, and physical accompaniment by CHWs was offered if necessary. Additionally, if the participant had a general practitioner, he/she was informed of the test results and referral process with the participant’s consent.

### 2.3 Involved partners

This project was implemented by the public service for the homeless in the Paris region (Samusocial de Paris) in partnership with the regional coordination committees for the fight against sexually transmitted infections and HIV (COREHIV) and community NGOs in the HIV sector. A more detailed description of the partners is available in Supplementary material.

### 2.4 Population targeted

All adult residents of the selected homeless hostels were invited to attend the workshops and RDT screening sessions. To target as many individuals as possible, the presence of children was permitted, and volunteer childminders were made available to provide care for them if needed. The workshops primarily targeted women, as they were the priority demographic within the hostel program and the population the most present in the hostels during daytime hours. However, with the agreement of the women who participated in the initial workshop, men were also welcomed to subsequent workshops.

## 3 Methods of feasibility and efficiency assessment

### 3.1 Design and sample

Data were collected in non-anonymized form with the patient’s consent, as it was necessary for physicians to have access to patient contact details (for example in the event of a positive test). However, patient data were later pseudonymized for analysis by replacing all patient-identifying variables with a unique ID number. This assessment of feasibility and efficiency combined qualitative and quantitative methods, including: the use of qualitative field observations, semi-directive interviews and focus groups with CHWs, STI screening results, quantitative indicators recorded at each workshop and satisfaction questionnaires for participants.

Quantitative indicators included: intervention characteristics (place; date; tools used; number of participants; health information provided); participant characteristics; tools used; characteristics of the patient screened (socio-demographic characteristics; health history; knowledge of sexual infection prevention methods), and potential referral performed following the workshop. Satisfaction assessment covering subjects including the reception they received, the selection of topics covered, the clarity of information provided, the effectiveness of the workshop tools, and the acquisition of new skills. Participant satisfaction was initially recorded using a paper questionnaire. However, from January 2023 onwards, due to writing difficulties reported by some participants, satisfaction surveys were carried out orally, then transcribed on paper by CHWs.

Qualitative field observations and quantitative indicators were systematically recorded by study investigators during each workshop. At the end of each workshop, satisfaction of all participants was asked. Focus groups were organized with CHWs in the middle at the end of the 8-week program timeline. Semi-structured interviews were conducted with all CHWs involved in the project between May and June 2022.

### 3.2 Procedure and materials

Throughout the program, quantitative indicators and participant satisfaction levels were systematically collected at the end of each workshop. Data collected by CHWs were then entered digitally and stored online via an accredited health data storage tool (WEPI 1.0). A short training session was organized with CHWs to introduce this software, and the study team was available at any time for questions or potential adaptations necessary for the questionnaire.

During the six first months of 2022, a researcher in public health and sociology conducted: six standardized semi-directive interviews with CHWs and 16 qualitative field observations using an observation grid. Each semi-directive interview was audio recorded and later transcribed word-for-word with the CHWs’ consent. These interviews focused on: (1) CHWs’ professional background and current tasks; (2) relationships with other health, social and medico-social professionals; (3) social support; (4) experience with organizing collective workshops; (5) and program strengths and weaknesses. Observations focused on: (1) the health education information provided by the CHWs during workshops, including the nature of the information and topics covered, CHWs’ knowledge and perspectives, vocabulary used, accessibility of the information provided, and adaptation to the participants’ literacy level; (2) the CHWs’ intervention approach, including their posture, communication style, tools used, emphasis on participant involvement and discussion; and (3) the residents’ level of knowledge and health literacy, including their experiences and practices.

Outside of this period, qualitative field observations were carried out during each workshop and screening sessions by a member of the study team. During the observations, all drivers of and barriers to program success were noted. These observations were recorded in a notebook and discussed with the CHWs, project members and/or participants. Focus groups with CHWs were conducted in the middle and at the end of 8-week period. CHWs’ were solicited during focus groups to share their experience and feedback on their missions.

### 3.3 Measures

To assess program feasibility, the measures recorded included the number of workshop participants, their socio-demographic characteristics and their level of knowledge regarding STI prevention methods, as well as the topics discussed by CHWs. These indicators were supplemented with the comments of CHWs during semi-directive interviews, focus groups and systematic field observations, allowing us to identify the drivers of and barriers to program success.

To measure the program’s efficiency, the number of RTDs performed, the rate of positivity, number of referrals to healthcare services and satisfaction levels of participants were used. In addition, field observations and feedback from CHWs (focus groups and interviews) were used to identify direct benefits (on sexual health prevention) and ancillary benefits (on other topics) for participants.

### 3.4 Data analysis strategy

For quantitative data, data quality control was carried out monthly, and data reporting to CHWs was carried out at the end of each workshop session The data were analyzed with R version 4.3.0. Results are presented as the number and percentage for qualitative variables and mean and standard deviation for quantitative variables.

For qualitative data, we applied the seven steps of the framework method (38) designed for analysis of textual data set involving multi-disciplinary research team: (1) transcription; (2) familiarization with the interview; (3) coding; (4) developing a working analytical framework; (5) applying the analytical framework; (6) charting data into the framework matrix; (7) interpreting the data. Data analysis was achieved by cross-referencing common elements related to the drivers of and obstacles to the project’s feasibility and effectiveness. The interviews were read twice by two independent researchers to ensure consistency, and the quotations were classified under the different themes by consensus of the entire study team.

### 3.5 Ethics statement

Informed consent was obtained from all participants. Data were collected in non-anonymized form with the patient’s consent, as it was necessary for physicians to have access to patient contact details (for example in the event of a positive test). However, patient data were later pseudonymized for analysis by replacing all patient-identifying variables with a unique ID number. Data were collected by CHWs from accredited associations. The data was stored on software approved for the storage of health data. These measures ensure that participants’ rights and privacy were protected throughout the study. By adhering to these ethical principles, we preserve confidentiality, respect autonomy, and give priority to the well-being of the people involved in our research. The partner associations are accredited in accordance with the Order of 1 August 2016 of the Ministry of Social Affairs and Health setting out the conditions for carrying out RDT orientation for HIV infection, hepatitis B and hepatitis C in medico-social or associative settings.

## 4 Findings

**Figure 1:**
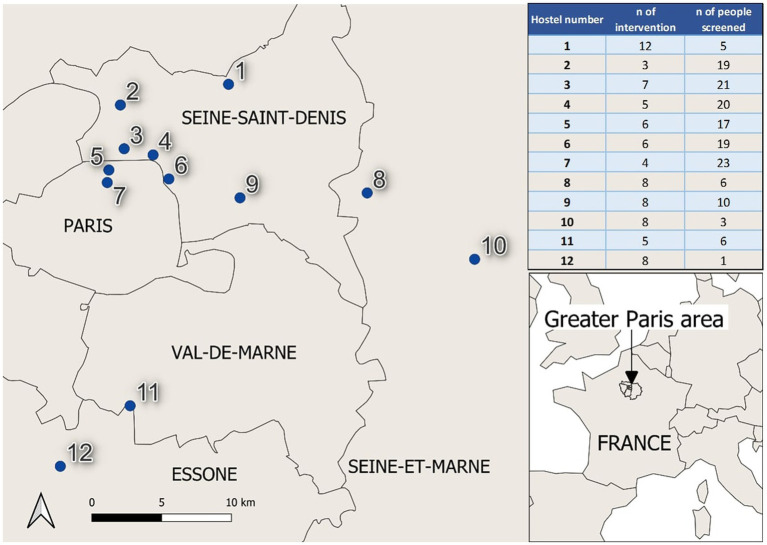
Homeless hostel location with number of workshops and STI screening and number of people screened n* = number.

**Figure 2:**
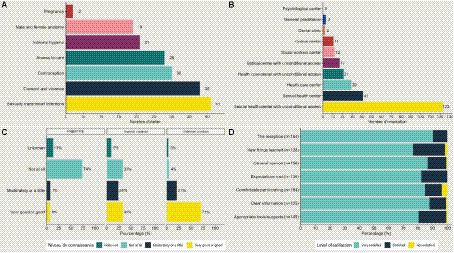
Quantitative indicators for program evaluation. **(A)** Themes addressed during workshops (*n* = 80). **(B)** Referrals performed following workshops (*n* = 80). **(C)** Level of knowledge of STI prevention methods (*n* = 150). **(D)** Level of satisfaction reported by the participants.

Between April 2021 and June 2023, a total of 80 outreach actions were conducted in the 12 homeless hostels targeted by our study. Actions included door-to-door canvassing (*n* = 15) and workshops (*n* = 65), with 53 workshops exclusively for women and 12 being mixed gender. The duration of each action ranged from 3 to 12 (non-consecutive) days, depending on the hostel concerned (Figure 1). The topics most frequently covered in workshops were STIs, consent and domestic violence, contraception methods and difficulties accessing care (Figure 2A).

### 4.1 Quantitative findings

In total, 542 women and 30 men participated in workshops for at least 1 day (individuals could be counted multiple times, as people may have attended multiple workshops during the session). On average, each workshop was attended by 9 individuals. Following the workshops, 123 individuals were referred to a center of STIs screening, 41 sexual health center with unconditional access, 29 to a general healthcare center and 28 individuals were referred to an ophthalmological care center with unconditional or free access. Other referral statistics are presented in Figure 2B.

During the 30 RDT screening sessions, a total of 150 individuals underwent testing for HIV, hepatitis B, and/or hepatitis C (Table 1). The majority of those tested were women (78.7%), with an average age of 36 years (35.5 for women, 38.9 for men). Most of individuals (>70%) completed all three RDT tests (HIV, hepatitis B, and hepatitis C). The positivity rate was 6.7% for hepatitis B, 0.9% for hepatitis C, and there were no HIV-positive test results. Almost half of the participants (48.7%) reported not being registered with a general practitioner. While 64.7% had consulted a medical professional at least once in the past year, 9.3% claimed to have never consulted a doctor. Within the last year, 12.6% of participants reported engaging in unprotected sex, and 9.3% experienced condom accidents. However, it is important to note that these rates were likely underestimates due to the sensitive nature of these questions. Approximately two-thirds of the participants (62.0%) had previously undergone screening for STIs, primarily within the last 3 years and in a hospital or laboratory setting. The primary reason cited for not undergoing screening was lack of opportunity. Knowledge of STI prevention varied from one preventive method to another: high for external condoms (71%), average for internal condoms (33%) and low for HIV pre- and post-exposure prophylaxis (PEP/PrEP) (6%) (Figure 2C). The percentage of individuals who reported having previously received serology testing was 34.0%.

**Table 1:** Characteristics of people screened, screening results, and risk behaviors (*n* = 150).

Variable	Level	Number	Percentage
Gender	Female	118	78.7	Male	26	17.3	Unknown	6	4.0
Age (year)	Mean and SD	36	9.5
General practitioner	Yes	76	50.7	No	73	48.7	Unknown	1	0.7
Time since lastConsultation with a generalPractitioner	<3 months	64	42.7	3 months-1 year	33	22.0	>1 year	29	19.3	Never	14	9.3	Unknown	10	6.7
Unprotected sexual relations	Yes	19	12.6
Condom accident	Yes	14	9.3
Oral-genital practice	Yes	7	4.7
Previous screening	Yes	93	62.0	No	46	30.7	Unknown	11	7.3
Time since last screening	Less than 1 year	24	25.8
	1 to 3 years	40	43.0
	More than 3 years	12	12.9	Unknown	17	18.3
Place of last screening	Hospital	50	53.8
	Laboratory	28	30.1
	Mobile unit	5	5.4	Other	2	2.1	Unknown	8	8.6
Serology	Yes	51	34.0	No	66	44.0	Unknown	33	22.0
Hepatitis B screening	Yes	105	70.0
Results	Positive	7	6.7	Negative	98	93.3
Hepatitis C screening	Yes	107	71.3
Results	Positive	1	0.9	Negative	106	99.1
HIV screening	Yes	146	97.3
Results	Positive	0	0.0	Negative	146	100.0

Between 135 (22%) and 165 (28%) people gave their opinion on the workshop, depending on the question. For each question posed, the majority of participants (>76%) reported being very satisfied. Overall, 86.5% of participants reported being very satisfied with the workshop and 12.8% satisfied (Figure 2D).

### 4.2 Qualitative findings

**Figure 3:**
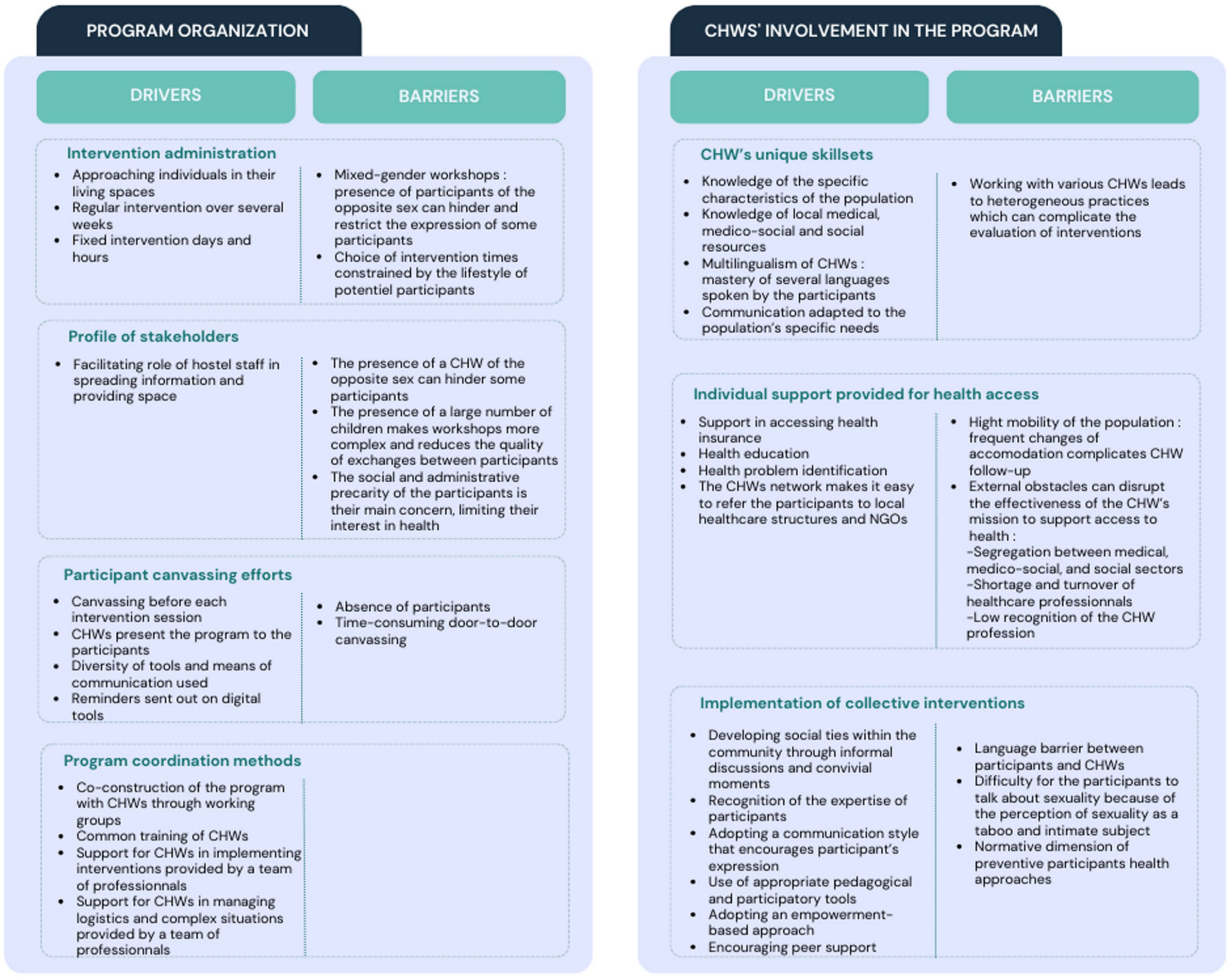
Key factors in the implementation and effectiveness of the program.

The thematic analysis of qualitative data enabled the identification of two axes that positively and negatively influence the feasibility and effectiveness of this program: program organization and involvement of CHWs (Figure 3).

#### 4.2.1 Program organization

Within the organizational axis, four sub-themes were identified as key factors for project implementation: intervention administration, stakeholder profile, canvassing, and program coordination methods.

Regarding intervention administration, engaging with individuals by approaching them in their living spaces, as well as the longitudinal nature of the program (actions conducted over several weeks) have been recognized by CHWs and investigators as essential factors for project effectiveness.

The profile of stakeholders was also important. Firstly, CHWs of the opposite gender sometimes created discomfort and stifled participant expression. Secondly, many participants were highly preoccupied with their complex social and administrative situations, and often considered health (especially sexual health) to be a secondary concern. Participants seized the opportunity to have qualified professionals in front of them to ask all their social and administrative questions, which sometimes made it difficult to bring the subject of sexual health back into the workshop.

Thirdly, in the absence of childcare facilities, children were also present at the hostel during the workshops, which CHWs and investigators reported negatively impacting the fluidity of the intervention. During observations, canvassing was highlighted as a key element for program success, functioning best when conducted by CHWs prior to program commencement and repeated before each action using diverse methods and tools.

Lastly, the coordination of the program by an operator external to the CHWs’ NGOs was underscored as an important success factor, particularly for achieving uniform practices, managing logistical challenges, and facilitating the training and support of CHWs.

#### 4.2.2 Involvement of CHWs

The involvement of CHWs in this project constituted the second axis. Within this axis, the first sub-theme focused on CHWs’ unique skillsets. The ability of CHWs to tailor their communication to the literacy levels and specific characteristics of the audience was highlighted by participants and investigators as a major driver of project effectiveness. This was facilitated by their understanding of the target audience and use of interview techniques, interactive activities, simplification of medical information, an empowerment-based approach, and various strategies used to alleviate discomfort related to the perception of sexuality as a taboo and intimate subject.

The second sub-theme was the individual support provided to participants by CHWs for better healthcare access. CHWs are trained in directing participants to local healthcare structures, helping them to overcome their lack of knowledge regarding healthcare system function. However, access to and effective use of healthcare facilities by individuals experiencing homelessness are sometimes hindered by external factors such as the segregation between medical, medico-social, and social sectors.

The third sub-theme focused on the implementation of collective actions, fostering social connections among participants, and addressing a strong need for social interaction in this isolated population. This program enabled participants to create social links, develop relationships and help each other. The observations during the workshops revealed that many participants did not know each other before the workshops despite living in the same hostel. During interviews, women reported that the workshops enabled them to meet new people. For example, one woman reported: “*We did not even know each other, not until we came here*.” Strengthening social ties was facilitated by informal CHW discussions and convivial moments such as shared meals at the end of the program. The program’s end was almost always perceived as unexpected by the participants (in the satisfaction survey, one woman reported “*I wish it would keep going*”), and after the final session participants shared their thanks and expressed their desire to continue. Some participants expressed the wish to take on responsibilities in the CHW’s NGO. This approach was encouraged by the CHWs and facilitated by the presence of a strong network of NGO community relays in the area.

BOX 1 **Who are the community healthcare workers in this study?**

Between May and June 2022, a total of 17 CHWs were recruited to participate in this study. To facilitate a deeper understanding of their profiles, six CHWs were interviewed using a standardized open questionnaire. These interviews focused on specific areas related to the study’s subject, including the CHW’s professional background and current missions. The data collected were analyzed by cross-referencing common discourse elements.

CHWs involved in this program were mainly born in West Africa, thus sharing the experience of immigrating to France with the target population. They were aged between 24 and 56 (average age 45) with varying levels of education: from high school baccalaureate to PhD. All of them received informal training in public health (e.g., training courses provided by NGOs) on several subjects: chronic illnesses, STI prevention, sexual violence, counseling, peer support, and therapeutic patient education. Despite their different professional backgrounds, they share a common professional experience close to HIV/AIDS and have generally been involved as employees or unpaid volunteers in the fight against the epidemic in France or African countries for several years.

## 5 Discussion

We developed, conducted and evaluated a CHW-led sexual health outreach program in homeless hostels in the greater Paris region. Our assessment determined that this program was highly feasible, reaching over 500 individuals belonging to a highly vulnerable and socially isolated population that is particularly challenging to reach through conventional health interventions. The effectiveness of this program was demonstrated by high rates of voluntary STI testing and referral to healthcare services, as well as through the improvement of sexual health literacy. Participants also reported broader impacts on wellbeing, such as the promotion of social connections and improved social support.

The involvement of CHWs emerged as a crucial determinant of this program’s success. Peer-to-peer exchanges (both between participants and between CHWs and participants) helped participants to express themselves and describe the problems they encounter, ultimately guiding them toward empowerment. Such positive impacts of CHW-led outreach programs are widely reported in the literature. Deep knowledge of local healthcare resources allows CHWs to support people in accessing healthcare, and by improving health literacy CHWs help to decrease loss to follow-up and addressing barriers to care (11, 15, 32, 39). CHWs are also trained to have strong communications skills, adapting their discourse and techniques to specific populations (11), enabling better identification and understanding of needs (13, 31), and providing assistance on social and health issues (31) by establishing links with NGO’s (40). The definition of collective workshops as forums and the approachable demeanor of CHWs facilitated exchange between participants and CHWs, which revealed strong health needs of this population, that are not specific to STIs but cover a broader spectrum of health issues, as illustrated by the large number of referrals to health care services made following workshops.

The implementation of outreach programs by CHWs using an empowerment-based approach is a key factor in the program’s success. However, certain obstacles remain, such as the lack of recognition of the CHW profession (although this is evolving), and fragmentation between the health, medico-social and social fields, referred by several research (11, 12, 17), which can make some support and referral between services difficult. Better integrating CHWs into more formal healthcare systems is known as a key factor in the effectiveness of this type of program (9, 12, 17, 31, 41–43).

To date, most outreach work targeting homeless populations in France has focused on individuals living on the street or accessing services such as food distribution. Our program was thus innovative in specifically targeting homeless populations housed, even if temporarily, in homeless hostels. We found that these people faced many of the same challenges and social difficulties often reported by those living in the street, including language barriers, difficulty accessing care, lack of knowledge about resources available and irregular immigration status. An advantage to targeting this population where they are housed is that it facilitated a combined outreach design, including the use of both door-to-door canvassing and regular WhatsApp reminders, helping to ensure a large number of participants.

Our outreach program had an indirect impact on participant health through the creation of social bonds. Social connectedness between peers has previously been identified as an important factor for improving healthcare use, physical health and overall wellbeing (13, 44, 45). The longitudinal aspect of the program, including regular workshops and STI screening sessions over an extended period, enabled the creation of trusting relationships between CHWs and participants, beneficial both within the framework of the project’s aims regarding sexual health outreach and more broadly in the fight against social isolation. CHW support also contributes to improving communication between community members on sensitive issues. For example, the issues of consent and sexual violence were often raised by participants, highlighting this as an important subject to explore further in this population. Further, fostering social ties may make it easier to bring health issues to the fore that are generally excluded from the priorities of these populations. Some of the individuals included in our study have great difficulty finding enough food to eat, have no access to employment or social housing, and in some cases it can take years to resolve administrative challenges, such as those associated with immigration. These combined factors underlie vulnerabilities in this population’s sexual health that justify setting up our program, but also explain why some homeless people may not perceive it as a priority, which can be a significant obstacle to reaching the target audience.

The program’s external coordination enabled us to manage all aspects of the project’s financing, logistics and evaluation. This centralization was essential to the smooth running and sustainability of the project, as has been highlighted in other community-based outreach programs (14, 30, 39, 46). Networking for this project took considerable time before its eventual implementation but was facilitated by a strong presence of collaborating NGOs and by the study team’s coordinating role in the region. The involvement of diverse partners helped to improve communication and collaboration and enriched the development and evaluation of the project. However, these diverse partners also have heterogeneous training and outreach practices, which can be a limitation for project efficiency. Group work sessions and common training courses are therefore essential to overcome these difficulties (17, 46).

### 5.1 Strengths, limitations, and perspectives

The design of this study enabled highlighting of the strengths and weaknesses of our outreach program (12). Several complementary methods (quantitative and qualitative) were used and different stakeholders were involved (interviewers, CHWs and participants), enabling us to cross-reference information and understand the impacts of the outreach program from diverse points of view.

However, the continued improvement of our interventions and evaluation tools is necessary, in particular the evaluation of satisfaction of study participants, which was hampered by limitations of written and spoken language, suggesting a need to design more inclusive and accessible tools. Into the future, program evaluation could be improved by using more pre- and post-intervention indicators to assess changes in knowledge and unsafe sexual behavior. Indeed, an appropriate next step would be to evaluate impacts of CHW interventions before, during and after the program’s implementation by taking longitudinal measurements of such indicators. However, such an evaluation is likely to face significant logistical hurdles, due to the transient nature of stays in homeless hostels and difficulty maintaining follow-up with the target population. In this sense, the flexible and non-compulsory nature of workshop attendance is both a strength, allowing participants to come and go as they please, and a limitation, posing challenges for program evaluation. Regarding indicators such as participant sexual health knowledge, it is also unclear which methods are most appropriate for intervention evaluation. For example, language barriers are a clear limitation for standardized questionnaire testing and peer influence poses clear challenges for group oral testing, while a lack of resources poses challenges for the conducting of repeated, standardized one-on-one interviews.

Our program could also potentially be improved by allowing participants to become more involved in program decision-making (17, 47). In addition, encouraging women to take a more proactive role toward their peers could be highly beneficial (30, 32, 45, 48). This could be the first step toward a real community health approach, helping them to exercise greater control over the decisions and actions that affect their health.

To extend the reach of this program across the tens of thousands of highly mobile people staying in homeless hostels in Paris, it seems appropriate to repeat these sessions in the same hostels at regular intervals but also to expand the program to areas not currently covered. Another key area for improvement in the future is to better accommodate children’s attendance during the workshop, to minimize interruptions and facilitate communication in a more intimate environment.

The question of gender, which has not been deeply investigated, occupies a central place in our reflections on the future of this program. The establishment of effective gender diversity comes up against the need to respect the specific needs of study participants. In particular, the assessment carried out prior to setting up the program highlighted the need to offer single-gender workshops in order to allow individuals to feel more comfortable expressing themselves in a group setting. While this program has been found to be effectiveness and useful for homeless women, an appropriate strategy for reaching homeless men who are not sufficiently involved in this program needs to be considered. Programs funded by UNAIDS have shown that motivating and encouraging heterosexual men and promoting programs (49) that meet their needs has a positive impact not only on their own health and wellbeing, but also positively impacts women’s sexual and reproductive health (48). Men tend to be less likely to seek care than women, even though they are more likely to engage in risky behavior (drug users, multiple partners) (49), highlighting the importance of outreach programs that better engage with men.

Our program also aimed to provide information on the use of HIV PrEP in high-risk women, in line with current recommendations (50). There was little to no awareness of its use in this population, and no PrEP referrals have been made to date. Efforts to promote this prevention method must continue, and consideration must be given to how to identify and approach women (e.g., through counseling) who may benefit most.

## 6 Conclusion

This CHW-led sexual health outreach program targeting homeless people suggests significant added value of this type of program in terms of improving health literacy, encouraging healthcare access and combating social isolation. The results of this study may provide guidance to stakeholders wishing to set up similar programs and encourage political decision-makers to develop such strategies among vulnerable populations. Finally, this project gives a voice to a group of people often overlooked in France, empowering them to address health and social challenges they may face. However, this program will not bring about lasting improvement in the socioeconomic situation of homeless people in extremely precarious situations, which can only be achieved through high-level government responses to their precarity, including housing and administrative reform. In the absence of such progress, these outreach programs are only a lifeline for people often excluded from mainstream services.

## Data availability statement

The datasets presented in this article are not readily available to protect the anonymization of individuals. Requests to access the datasets should be directed to Lison Ramblière (l.rambliere.research@gmail.com).

## Author contributions

EV: Project administration, Data curation, Formal analysis, Investigation, Methodology, Visualization, Writing – original draft, Conceptualization, Validation. LR: Data curation, Formal analysis, Methodology, Software, Visualization, Writing – original draft. MD: Project administration, Supervision, Writing – original draft. HL: Investigation, Project administration, Writing – review & editing. SG: Writing – review & editing, Project administration, Supervision. AP-C: Funding acquisition, Project administration, Supervision, Writing – review & editing. SR: Project administration, Supervision, Writing – review & editing. JG: Project administration, Writing – review & editing. WR: Writing – review & editing. EB: Project administration, Supervision, Writing – review & editing. MP: Funding acquisition, Project administration, Supervision, Writing – original draft.
