# Supplementary material for: Proof of concept of a sexual health outreach program led by community health workers in homeless hostels in the greater Paris region
Source: Front Public Health. 2024 Jan 12;11:1305874. doi: 10.3389/fpubh.2023.1305874 (PMC10811606; doi:10.3389/fpubh.2023.1305874)
Supplement: Supplementary file 1 [file Data_Sheet_1.DOCX]

**Supplementary** **materials**

Supplementary 1: Short description of the patterners involved in the program

The Samusocial of Paris played multiple roles in the program, including: 1) homeless hostel contacts and information, 2) coordinating efforts among all partners involved in health, housing, and social services, 3) providing financing and logistical support, 4) facilitating the work of CHWs by alleviating their constraints, and 5) conducting program evaluation and communication. During each intervention, one or more professionals were present to assist CHWs with mobilization, setup, and implementation processes.

The COREVIH is a national professional network coordinating public health action in the fight against STIs [(51)](https://sciwheel.com/work/citation?ids=15004241&pre=&suf=&sa=0&dbf=0). This network is deployed by region and then by locality. Each branch of COREVIH linked to a referral hospital, were set up to provide comprehensive care for HIV-positive patients through the following goals [(52)](https://sciwheel.com/work/citation?ids=15004292&pre=&suf=&sa=0&dbf=0): coordinating professionals; improving the quality and safety of care for people living with HIV/AIDS and harmonising practices; collecting and analysing epidemiological data; and participating in the evaluation of national programming to combat STIs. The COREVIHs draw on the expertise of local associations and their CHWs, providing local information, screening and referral services. In this program, the organization provides: 1) contacts with partner associations, 2) scientific support for data analysis, 3) communication around the project, 4) referral stand for positive HIV, HBV or HCV tests, and 5) assistance with the creation of educational tools.

The NGOs involved in the program are part of the COREVIH network and aim to improve the care and health of vulnerable people. They offer holistic support to migrants and people with chronic pathologies, to help them access rights and care. Most of them have expanded their initial scope of action on the fight against HIV/AIDS to include the promotion of sexual health and other chronic pathologies. The NGOs are made up of medical, medico-social, social, legal and occupational integration professionals, including CHWs, and provide a wide range of services: information and referral committee room, outreach prevention and screening activities, therapeutic education and peer support workshops, activities and social events. The approach used and the knowledge of the public and local operators were key factors in choosing NGOs and setting up the partnership.
